# Appropriate Use and Operationalization of Adherence to Digital Cognitive Behavioral Therapy for Depression and Anxiety in Youth: Systematic Review

**DOI:** 10.2196/37640

**Published:** 2022-08-17

**Authors:** Sophie H Li, Melinda R Achilles, Aliza Werner-Seidler, Joanne R Beames, Mirjana Subotic-Kerry, Bridianne O'Dea

**Affiliations:** 1 Black Dog Institute and School of Psychology The University of New South Wales Randwick Australia; 2 Black Dog Institute The University of New South Wales Randwick Australia

**Keywords:** adherence, youth, digital, cognitive behavioral therapy, review, mobile phone

## Abstract

**Background:**

Digital, self-guided cognitive behavioral therapy (CBT) interventions circumvent many barriers to in-person therapy for young people (aged 12-24 years), although adherence to these interventions is low. The absence or insufficient disclosure of recommendations or instructions for appropriate use may account for this. As such, many young people may not self-administer these interventions appropriately or receive the optimal degree of treatment.

**Objective:**

This systematic review aims to synthesize the literature on digital CBT for depression and anxiety in young people to describe how appropriate use has been defined and communicated to users as instructions for use, to describe how adherence has been measured, and to determine the associations between adherence and treatment outcomes.

**Methods:**

A systematic review was conducted with 2 reviewers (SHL and MRA) extracting data independently. Overall, 4 electronic databases (Embase, MEDLINE, PsycINFO, and Cochrane Library) were searched in April 2021 for studies that met the following inclusion criteria: participants aged between 12 and 24 years, evaluated a digital CBT intervention targeting depression or anxiety, and reported instructions or recommendations for use or measures of adherence. Studies that evaluated non-CBT interventions or cognitive- or behavioral-only interventions were excluded. Methodological quality was assessed using the Cochrane Risk of Bias Tool and the Integrated Quality Criteria for the Review of Multiple Study Designs.

**Results:**

There were 32 manuscripts that met the inclusion criteria, of which 28 (88%) were unique studies (N=16,578 youths). Definitions of appropriate use varied among the different interventions in terms of intended recipients, duration and frequency of use, and the features used to support engagement and adherence to appropriate use definitions. Reporting of appropriate use definitions in studies was inconsistent, with no study systematically describing components of appropriate use or providing information on how recommendations for use were relayed to users. Most often, definitions of appropriate use were derived from the study protocol and descriptions of intervention features. Adherence was mostly operationalized as the degree of intervention completion; however, reporting of adherence data was heterogeneous. There was little evidence of an association between degree of use and outcomes in the 9 studies that examined this.

**Conclusions:**

Definitions of appropriate use are unique to each digital CBT intervention. However, statements of appropriate use are not systematically reported in the literature. Furthermore, the extent to which recommendations for use are communicated to users is not routinely reported. Despite unique definitions of appropriate use, adherence was most often generically operationalized as the degree of intervention completion and was not consistently associated with outcomes. We proposed a framework to promote systematic reporting of definitions of appropriate use for digital interventions to provide guidance to users and to assist the development of appropriate and nuanced measures of adherence.

**Trial Registration:**

PROSPERO CRD42020208668; https://tinyurl.com/4bu2yram

## Introduction

### Background

Cognitive behavioral therapy (CBT) is a structured, skills-based psychotherapy, typically delivered in-person by a trained clinician over a duration of 12 to 18 weeks [1,2]. It is the gold standard psychological treatment for anxiety and depression [3] in both adults and young people, defined here as people aged between 12 and 24 years, consistent with the Australian Institute of Health and Welfare [4-7]. To safeguard the integrity of CBT and ensure that efficacy is maintained [8], CBT manuals provide clinicians with explicit instructions on administration. These manuals detail indications of who will benefit, the treatment targets and goals, the number and duration of sessions required, sequence of content delivery, and between-session practice and real-world enactment of skills (ie, homework [9]). Clinicians also adapt various engagement strategies to promote adherence to CBT, including developing a therapeutic alliance through collaborative goal setting, communicating and enhancing treatment expectancies, and clarifying concepts to ensure that treatment processes and rationale are well understood [1,2,5-7,10,11]. Some CBT manuals also differentiate the critical, core treatment components from optional modules and strategies to measure symptoms and treatment outcomes [1]. To summarize, the components of CBT manuals that guide clinicians’ administration as intended are as follows: recipients, target condition, number and duration of sessions, sequence of content, homework activities, engagement-promoting strategies, core therapeutic components, assessment and monitoring, and crisis management.

Despite its efficacy, the uptake of in-person CBT is suboptimal across all ages [12-15]. In young people, this is due in part to the affordability and availability of trained practitioners, perceived stigma, poor mental health literacy, and a preference for self-reliance [16-18]. Among the young people who do seek help, most do not receive CBT [19,20]. Improving access and uptake of CBT is particularly critical for young people, as three-quarter of depression and anxiety cases emerge by late adolescence [21], and these disorders are a leading cause of disability in this age group [22,23]. The transition of CBT from in-person professional administration to digital, self-directed delivery was hypothesized to overcome many of the treatment barriers faced by young people [24]. A recent systematic review and meta-analysis of internet- and computer-delivered CBT for youth confirmed the positive effects of these interventions on symptom reduction [25]. However, adherence to many of these interventions is low despite the alignment of digital CBT with young people’s help-seeking preferences [24].

Although personal factors such as motivation may account for low adherence, reliance on the young person to appropriately self-administer digital CBT interventions with none to limited clinician guidance or supervision may also contribute. Thus, similar to any self-administered treatment, it is essential that the young person is provided with clear instructions to ensure appropriate use; that is, to ensure that the intervention is used in a way that generates optimal clinical benefit. Unlike in-person CBT, where instructions for engaging with the treatment are well documented within manuals and directly relayed to youth through their clinician, it is unclear whether instructions or recommendations for the appropriate use of digital CBT are provided to users or consistently documented in the literature. Instructions for use may be provided during intervention onboarding or guided via explicit or implicit software design features. For example, many digital CBT interventions have used the design feature, “tunnelling,” to replicate the structure of in-person CBT, whereby therapeutic modules are presented sequentially to guide users through the content in an appropriate sequence [26,27]. Other software design features, such as tailoring content in response to user input, automated feedback, rewards and encouragement, and reminders and notifications, have also been used to replicate clinician guidance and supervision [28]. An understanding of how digital CBT interventions define appropriate use and how instructions for use are relayed to youth requires examination to determine their adequacy in supporting appropriate self-administration.

In addition to ensuring optimal outcomes, clear instructions for the use of digital CBT interventions are required for researchers and clinicians to operationalize users’ adherence to these interventions. Adherence is defined as a meaningful measure of the extent to which individuals’ intervention use corresponds with creators’ recommendations, instructions, and expectations of appropriate use [29,30]. For example, if the recommendation is to complete all modules within a digital intervention, adherence is measured by determining the number of modules completed. Alternatively, if the recommendation is to engage with a particular intervention activity on a specified occasion (eg, complete a mindfulness mediation upon waking), adherence is measured by determining the frequency of this event. In this way, adherence provides a measure of the validity of treatment administration. Thus, clear instructions for appropriate use and corresponding adherence measures are crucial for ensuring that the necessary standards of quality, safety, and efficacy are met. Despite the importance of this, in a review of the literature, Christ et al [25] found that many studies on digital CBT for youth generically operationalized adherence as program completion, heterogeneously reported as the proportion of participants who completed all treatment modules or the average number of modules completed across the sample. Several studies failed to report any adherence data, and definitions of appropriate use were not examined in the study by Christ [25].

Similar variability in the reporting of adherence has been found in reviews of adult digital health interventions. One systematic review found that almost three-quarter of studies (45/62, 73%) operationalized adherence as the degree of intervention use, reported as the number of completed modules and activities, log-ins, or time spent in the intervention [31]. Furthermore, statements on instructions for use were only reported in one-third (23/62, 37%) of studies, giving little indication of how interventions defined appropriate use [31]. Inadequate reporting of instructions for use and significant heterogeneity in reporting of adherence are further demonstrated in at least 5 systematic and narrative reviews of adherence to digital psychological interventions in adults [32-36]. Collectively, adherence appears to be operationalized in the absence of adequately reported definitions of appropriate use or is generically operationalized as adherence to total intervention exposure, based on the notion of “the more, the better” rather than explicitly measuring the accordance between recommended use and actual use. Studies have provided little justification for this approach [31]. Furthermore, studies of adults and youth have not consistently supported a linear relationship between adherence and outcomes [25,29,37-39].

Despite the prevalence of digital CBT interventions for youth [25], little is known about how young people have been instructed to use these interventions, how adherence to interventions has been operationalized, or whether measures of adherence adequately determine differences between actual and recommended use. It is important to examine this, as young people have different patterns of engagement with technology, preferences, and expectations compared with adults [40-42]. Clearer expectations of use may not only improve the effectiveness of digital CBT but also assist young people in selecting interventions most suited to their circumstances, while also facilitating greater endorsement and dissemination by mental health professionals. Establishing protocols for the disclosure of appropriate use and the operationalization of adherence is imperative for improving the uptake, adherence, and effectiveness of digital CBT among youth.

### Objectives

The primary aim of this systematic review was to synthesize the published literature on digital CBT interventions for depression and anxiety in young people to investigate the definitions of appropriate use and the disclosure of instructions for use to users. To this end, descriptions, recommendations or instructions of appropriate use, or intervention features guiding use were extracted from the included studies and mapped onto the 10 components within CBT manuals that typically guide administration as a means of determining definitions of appropriate use. A specific focus on digital CBT differentiated our review from recently published reviews on engagement in digital health interventions that incorporated CBT and non-CBT interventions. This review also aimed to examine how adherence to recommended use has been operationalized and measured and to determine the associations between digital CBT intervention use and outcomes among young people. This information will improve our understanding of how young people have been instructed to self-administer digital CBT interventions and will determine whether the measures of adherence used by researchers have accurately captured the degree to which young people complied with instructions for use.

## Methods

### Protocol and Registration

PRISMA (Preferred Reporting Items for Systematic Reviews and Meta-Analyses) was used to ensure the quality and consistency of the procedure and reporting [43]. The review protocol was registered with PROSPERO (CRD42020208668). We deviated from the original protocol to exclude cognitive- and behavioral-only interventions and mindfulness-based and acceptance and commitment therapy interventions to confine the focus to digital CBT. In addition, we found that the study manuscripts rarely reported on the instructions of appropriate use. We acknowledge that this information may have been provided to users within the intervention itself, but this is not clear. As such, we derived the information on appropriate use from the study protocols and intervention features.

### Ethical Considerations

Conducting a systematic review or peer-reviewed literature is not listed as a research activity that requires human ethics approval by the UNSW Research Ethics Board. As such, ethics approval was not applied for.

### Eligibility Criteria

#### Participants

Participants were young people aged between 12 and 24 years. This age range was selected to cover the full spectrum of youth, as defined by the Australian Institute of Health and Welfare [4]. Where the age range of participants extended 12 to 24 years, studies were excluded if the total sample contained <80% of participants in the target range. In the absence of age range data, a judgment was made based on the recruitment setting (eg, secondary school) and the reported mean age. Diagnostic status was not used as an eligibility criterion for samples.

#### Interventions

Eligible interventions included those directly targeting anxiety or depression (including transdiagnostic interventions) via a predominantly CBT-based psychological treatment delivered by a computer, smartphone, or internet platform. Interventions were also required to be used on more than one occasion. Purely cognitive or behavioral interventions alone were not included, nor were treatments aimed solely at problem-solving. Interventions that were not predominantly CBT, such as mindfulness-based interventions, acceptance and commitment therapy, and interpersonal psychotherapy, were excluded. Nonpsychological interventions, including exercise or physical activities, music, and art therapy, were excluded. Gratitude-based therapies and journaling were also excluded.

#### Comparison Groups

No restriction was imposed on the type or use of control or comparison groups.

#### Outcomes

Primary outcomes were symptoms of depression or anxiety measured using standardized, validated, and reliable instruments or scales, suitable for adolescents. Included studies were required to report at least one measure of adherence and any of the following: descriptions, statements, or instructions for appropriate use or recommendations of use; descriptions of intervention features supporting appropriate use, including adherence-promoting features; or the association between adherence and depression outcomes and adherence and anxiety outcomes.

#### Studies

Studies were included if they were written in English, published in peer-reviewed journals, and published after January 1, 1991. Studies were not excluded based on study type or quality; both controlled and uncontrolled studies (eg, pre-post studies without a control group) were included. Case studies were excluded.

### Search Strategy

#### Overview

The electronic databases, Embase, MEDLINE, PsycINFO, and Cochrane Library, were searched for articles published from January 1, 1991, to April 7, 2021. The start date was selected to coincide with the year in which the World Wide Web was introduced. The following search terms were used in the title, abstract, and keywords: (adolescent* OR *youth* OR child* OR teen* OR *young adult)* AND *(online* OR *digital* OR *internet* OR *app* OR *mHealth* OR *eHealth* OR *web* OR *web-based* OR *smartphone* OR *smart phone* OR computer*) AND (anx* OR depress* OR *affect* OR *mood)* AND *(cognitive therapy* OR *cognitive behavioural therapy* OR *cognitive behavior therapy* OR *cognitive behavioral therapy* OR *CBT)*. Additional sources were included through a hand search that comprised examining reference lists of key articles and systematic reviews and authors’ knowledge of manuscripts related to digital CBT for youth. Furthermore, the details from included studies were used to conduct a search to identify relevant manuscripts reporting on secondary or adherence outcomes from the included studies’ data sets.

#### Data Extraction and Synthesis

Articles were identified from the search strategy, and after duplicates were removed, titles and then abstracts were reviewed for relevance by the first author (SHL). A second reviewer (AWS) independently screened 10% of the identified studies to ensure the reliability of the eligibility criteria. Full texts of the remaining studies (n=174) were appraised by 2 reviewers (SHL and MRA) to determine eligibility. Reasons for exclusion were discussed, and consensus for eligibility was confirmed for 6 articles. In cases where a consensus between the 2 reviewers could not be reached, a third reviewer (MSK) assessed for eligibility and a consensus was reached. The 2 reviewers (SHL and MRA) independently performed the data extraction using a data extraction template (Multimedia Appendix 1) designed to identify information, including the following: study details, sample details, intervention details, and outcomes of interest. It is worth noting that, for delivery mode, interventions were categorized as *sequential* where modular content was presented sequentially or explicitly delivered in a sequential order with content only becoming available when preceding modules were completed, or *nonsequential* where content was unrestricted allowing complete self-navigation.

Study outcomes of interest included the following: descriptions, statements, or instructions of appropriate use for young people; measures of adherence; and associations between adherence and symptom outcomes. We relied only on the information provided in the study manuscripts. Using the components of manualized CBT, we identified information on appropriate use pertaining to the following: intended recipient of the intervention, intended target condition, intended number of modules to be completed, intended duration and frequency of use, instructions regarding real-world enactment of skills (homework), adherence-promoting features (embedded within the intervention), core components, and symptom assessment and monitoring. We also extracted data on when and how users were recommended to access in-person support, excluding risk management procedures that constituted the study protocol. In lieu of specific descriptions of appropriate use, we extracted any information on intervention design features or other study details that implied appropriate use. This included the following: inclusion and exclusion criteria to indicate intended recipients, duration of intervention access to indicate intended duration of use, and tunneling to indicate intended sequence of content use. The sources of information used to derive aspects of appropriate use were recorded (trial protocol, feature description, or a statement in the study manuscript). Post hoc determination of the number of modules needed to achieve benefits was not considered as constituting any aspect of appropriate use.

Adherence-promoting features included those explicitly used by the authors to improve adherence or those that fit within the following categories: supported use defined as use that involves support, encouragement or guidance from a person, reminders, rewards, gamification, social or peer support, tailoring defined as the capacity to tailor content to meet an individual’s requirements, personalized feedback including system-generated personalized feedback, customization of visual features such as avatars and color schemes, and interactive content defined as content requiring active inputs from the user. These categories were derived from past literature that endorsed supported use as an effective adherence promoter [44] and systematic reviews of user preferences [24,45]. Supported use was further categorized into autonomous, supported, or intervention-led blended, using the definitions provided by Fairburn and Patel [46] (Table 1). Interventions delivered in schools were categorized as supported, unless otherwise specified, and studies that did not specify support in the use of the intervention were recorded as autonomous. Interactive content was further coded as follows: activities, quizzes, homework activities, and multimedia content. The same intervention delivered under different conditions (eg, with or without additional in-person therapy sessions) was treated as 2 separate interventions and is presented in different rows in the tables. It is worth noting that, we elected to categorize personal feedback from a clinician or support person as supported use, not tailoring, as supported use is likely to have an element of personalization that does not necessarily involve tailored delivery of the intervention content. In addition, the included studies were screened for references to registered or published protocols. Where protocols were identified, they underwent the same data extraction and synthesis procedure as the study manuscripts to supplement data on descriptions, statements, or instructions for appropriate use. Finally, the number of aspects of digital self-administration described in each study was tallied to show the degree to which aspects of self-administration were disclosed.

**Table 1:** Characteristics of included studies (n=32).

Study and year published	Country, setting, and year conducted	Study design	Participants (intervention)			
			Population and setting	Sample size, n	Girl or woman (transgender person), %	Age (years), mean (SD; range)
Berg et al [47], 2020	Sweden, 2018	2×2 factorial design	Clinically relevant anxiety symptoms and comorbid depression	120	81	16.97 (1.20; 15-19)
Bevan Jones et al [48], 2020	United Kingdom	Pre-post feasibility trial	History or risk of depression	35	79	16.3 (2.36; 13-23)
Calear et al [49,50], 2009 and 2013	Australia, 30 schools, 2006	Cluster RCT^a^	Universal sample of secondary school students (30% with prior history of depression)	559	60	14.34 (0.75; 12-17)
Clarke et al [51], 2009	United States, health maintenance organization	RCT	History of depression or risk of depression	83	81	22.6 (2.3; 18-24)
Fleming et al [52], 2012	New Zealand, 2009-2010	RCT	Excluded from mainstream education	20	44	14.9 (0.79; 13-16)
Ip et al [53], 2016	Hong Kong, 3 schools, 2013-2015	RCT	Mild or moderate depressive symptoms	130	68.1	14.63 (0.81; 13-17)
Jaycox et al [54], 2019	United States, 5 schools, year undisclosed	Pre-post open trial	In 4 out of 5 schools, students were identified by school counselors and social workers; in the fifth school, all students in the health class participate.	51	56.9	15.02 (1.86; range NR^b^)
Kuosmanen et al [55], 2017	Ireland, Youthreach Centers, 2015-2016	Cluster RCT	From a second-chance education intervention. No symptom eligibility criteria	30	53.4	17.60 (1.24; 14-20)
Lenhard et al [56], 2017	Sweden, 2014-2015	12-week, single-blinded RCT	Obsessive compulsive disorder	33	46	14.6 (1.71; 12-17)
Lillevoll et al [57], 2014	Norway, 4 schools, 2009	4-arm RCT	No symptom eligibility criteria	527 (3 arms received intervention)	50	16.8 (1.0; 15-20)
Lucassen et al [58], 2020	New Zealand, 2014	Open trial—secondary analysis of 5 years of SPARX^c^ usage data	No symptom eligibility criteria	9079	65.7 girls, 2.3 transgender person	NR (NR; 12-19
March et al [26], 2018	Australia, 2014-2016	Open trial	Elevated anxiety	4425	66.39	12.95 (2.97; 7-17)
Melnyk et al [59], 2015	United States, large public university, 2012-2013	RCT	No symptom eligibility criteria	82	86.4	18.4 (1.9; range NR)
Merry et al [60], 2012	New Zealand, 12 primary health care youth clinics, general practices, and school-based counseling services	Randomized controlled noninferiority trial	Depressive symptoms	94	65.7	15.55 (1.54; 12-19)
O’Connor et al [61], 2020	Canada, 2014-2016	2-arm, multisite, pilot RCTs	Anxiety concerns	36	90	15.3 (1.2; 13-17)
O’Dea et al [62], 2020	Australia, 2018-2019	RCT	No symptom eligibility criteria	94	86.5	14.82 (0.93; 12-16)
O’Kearney et al [63], 2009	Australia, girls-only schools	Controlled trial	No symptom eligibility criteria	67	100	Year 10
Radomski et al [64], 2020	Canada, 2016-2018	RCT	Mild to moderate anxiety	258	71	16.6 (1.7; 13-19)
Smith et al [65], 2015	United Kingdom, schools, 2011-2013	RCT	Significant depression	55	NR	NR (NR; 12−16)
Spence et al [66], 2011	Australia, 2006-2008	3-arm RCT	Anxiety disorder	44	59.13	13.98 (1.63; 12-18)
Stallard et al [67], 2011	United Kingdom, home and school	RCT	Anxiety disorder or mild to moderate depression	10	NR	13.5 (NR; 11-17)
Stasiak et al [68], 2012	New Zealand, 8 urban high schools	RCT	Low mood	17	41	15.2 (1.5; 13-18)
Stjerneklar et al [69], 2019	Denmark, 2015-2017	RCT	Anxiety disorder	33	79	15 (1.3; 13-17)
Tillfors et al [70], 2011	Sweden, 2007-2008	RCT	Social anxiety disorder	10	89	16.5 (1.6; 15-21)
Van Voorhees et al [71], 2009	United States, 2007	RCT	Subclinical depression from primary care sites	43	77	17 (NR; NR)
Van Voorhees et al [72], 2020; Gladstone et al [73,74], 2018 and 2020	United States, 2012-2016	RCT	Subclinical depression from 31 primary care sites	193	68	15.4 (1.5; 13-18)
Whittaker et al [75,76], 2017 and 2012	New Zealand, 15 schools, 2009-2011	RCT	No symptom eligibility criteria	426	68	14.3 (undisclosed; 13-17)
Wuthrich et al [27], 2012	Australia	RCT	Anxiety disorder	24	62.79	15.17 (1.11; 14-17)

^a^RCT: randomized controlled trial.

^b^NR: not reported.

^c^SPARX: Smart, Positive, Active, Realistic, X-factor thoughts.

#### Methodological Quality Assessment

The methodological quality of the included studies was assessed using the Cochrane Risk of Bias (ROB) Tool for randomized controlled trials (RCTs) [77] and the Integrated Quality Criteria for the Review of Multiple Study Designs [78] for other study designs. Quality ratings were determined independently by 2 reviewers (SHL and JRB) and then compared to resolve differences. Studies were considered methodologically sound if they met the Integrated Quality Criteria for the Review of Multiple Study Designs criteria for inclusion [78] or were rated as low in the ROB overall assessment of bias.

## Results

### Overview of Included Studies

#### Overview

**Figure 1:**
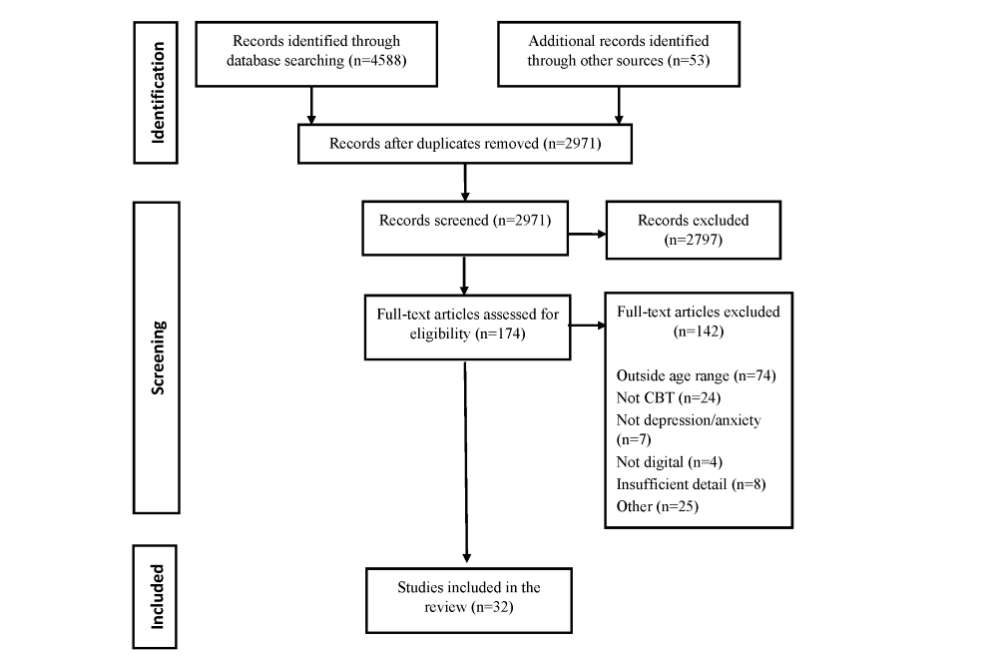
PRISMA (Preferred Reporting Items for Systematic Reviews and Meta-Analyses) flow diagram.

The search resulted in 4641 studies, of which 174 full-text articles were identified. A total of 32 studies met all inclusion criteria and were included in the systematic review. The screening process and reasons for exclusion are presented in Figure 1. Table 1 summarizes the study, sample, and intervention characteristics of the 32 included manuscripts. On 3 occasions, >1 manuscript was produced from a single study; as such, these manuscripts were reported together, leaving a total of 28 independent studies.

#### Study Design and Country of Origin (Setting)

Of the 28 studies, most were RCTs (n=22, 78%), and the others were open trials (n=3, 10%), feasibility trials (n=11, 39%), 1 (3%) non-RCT, and 1 (3%) factorial design. The majority were conducted in Australia and New Zealand (11/28, 39%), Europe and the United Kingdom (9/28, 32%), and the United States (5/28, 17%), with others in Canada (2/28, 7%) and Hong Kong (1/28, 3%).

#### Sample Characteristics

Across the 28 studies, 16,578 participants received the intervention. Sample sizes varied considerably among studies, ranging from 10 to 9079 (mean 592.07, SD 1857.44; median 61). Overall, 42% (12/28) of studies had sample sizes <50, and 57% (16/28) had sample sizes >50. Regarding gender representation, 75% (21/28) of studies had >55% of their sample as girls or women, 10% (3/28) of studies had between 45% and 55% of the sample girls or women, 7% (2/28) of studies had <45% of the sample as girls or women, and 7% (2/28) of studies did not disclose gender representation of their samples. The girl or woman representation within the samples ranged from 41% to 100%. Across all studies, the mean age was 15.74 years (range 12.95-22.60), with a total age range of 7 to 24 years. A total of 10% (3/28) of studies did not report the mean age of the participants, and 14% (4/28) of studies did not report the age range.

#### Intervention Characteristics

Intervention characteristics are presented in Table 2. A total of 20 interventions from 28 studies were identified and included in the review, with the same intervention used in several studies: SPARX (Smart, Positive, Active, Realistic, X-factor thoughts; University of Auckland; n=4), MoodGYM (Australian National University; n=3), Brave Online (University of Queensland; n=2), CATCH-IT (University of Illinois; Competent Adulthood Transition with Cognitive Behavioral Humanistic and Interpersonal Training; n=2, not including the modified version), and Breathe (University of Alberta; Being Real, Easing Anxiety: Tools Helping Electronically; n=2). A total of 28% (8/28) of interventions targeted depression, 25% (7/28) targeted depression and anxiety, 10% (3/28) targeted unspecified anxiety, and 7% (2/28) targeted a specific anxiety disorder (obsessive compulsive disorder and social anxiety disorder). The digital delivery formats included web-based (n=11), computer or CD-ROM (n=6), multiplatform (n=1), weblinks viewed on a smartphone (n=1), and 1 smartphone app (n=1). Several interventions included elements of other therapeutic approaches in addition to CBT, such as interpersonal therapy (2/28, 7%), family systems therapy (1/28, 3%), and positive psychology (1/28, 3%). The total number of modules ranged from 4 to 15 (mean 8.15, SD 2.74). A total of 10% (3/28) studies did not specify the number of modules contained in the intervention. Most interventions (14/20, 70%) were sequential, 10% (2/20) were nonsequential (WeClick and MoodHwb), and 20% (4/20) were undetermined.

**Table 2:** Characteristics of interventions (n=20).

Study and year published	Intervention name	Target condition	Delivery mode	Delivery format	Therapeutic model	Number of modules or sessions
Berg et al [47], 2020	Undisclosed	Anxiety and depression	Web	Sequential	CBT^a^	8 modules
Bevan Jones et al [48], 2020	MoodHwb	Depression	Multiplatform (web, app)	Nonsequential	CBT, IPT^b^, positive psychology, family systems therapy	Unclear
Calear et al [49,50], 2009 and 2013; Lillevoll et al [57], 2014; O’Kearney et al [63], 2009	MoodGYM	Anxiety and depression	Web	Sequential	CBT	5 modules, 29 exercises
Clarke et al [51], 2009	Undisclosed	Depression	Web	Undisclosed	CBT	Unclear; 4 components
Fleming et al [52], 2012; Kuosmanen et al [55], 2017^c^; Lucassen et al [58], 2020; Merry et al [60], 2012	SPARX^c^	Depression	Computer	Sequential	CBT	7 modules
Ip et al [53], 2016^d^	Grasp the opportunity (translated to Chinese and modified from CATCH-IT)	Depression	Web	Undisclosed	CBT	10 modules
Jaycox et al [54], 2019	The LIFT program	Anxiety and depression	Computer	Sequential	CBT	7 modules
Lenhard et al [56], 2017	BIP OCD^e^	OCD	Web	Undisclosed	CBT	12 modules
March et al [26], 2018; Spence et al [66], 2011	BRAVE Online	Anxiety	Web	Sequential	CBT	10 modules
Melnyk et al [59], 2015	COPE^f^	Anxiety and depression	Web	Sequential	CBT	7 modules
O’Connor et al [61], 2020; Radomski et al [64], 2020	Breathe^g^	Anxiety	Web	Sequential	CBT	8 modules; 6 modules^h^
O’Dea et al [62], 2020	WeClick	Depression, anxiety	Smartphone app	Nonsequential	CBT	4 character stories
Smith et al [65], 2015	Stressbusters	Depression (mild-moderate)	Computer	Sequential	CBT	8 modules
Stallard et al [67], 2011	TFD^i^	Anxiety, depression	CD-ROM	Undisclosed	CBT	6 modules
Stasiak et al [68], 2012	The Journey	Depression	CD-ROM	Sequential	CBT	7 modules
Stjerneklar et al [69], 2019	ChilledOut Online (Danish)	Anxiety, depression	Web	Sequential	CBT	8 modules
Tillfors et al [70], 2011	Undisclosed	Social anxiety disorder	Web	Sequential	CBT	9 modules
Van Voorhees et al [71,72], 2020 and 2009; Gladstone et al [73,74], 2018 and 2020^j^	CATCH-IT	Depression	Web	Sequential	CBT and IPT	14 modules; 14 modules (plus 1 optional anxiety module and 5 parent modules)
Whittaker et al [75,76], 2017 and 2012	MEMO CBT	Depression	Web links viewed on smartphone	Sequential	CBT	2 messages daily, for 9 weeks
Wuthrich et al [27], 2012	Cool Teens	Anxiety	CD-ROM	Sequential	CBT	8 modules

^a^CBT: cognitive behavioral therapy.

^b^IPT: interpersonal psychotherapy.

^c^SPARX-R is a revised version of the original Smart, Positive, Active, Realistic, X-factor (SPARX) intervention. The content of SPARX-R is essentially the same; however, it is framed as a preventive intervention for young people who *feel down, stressed, or angry* rather than focusing exclusively on depression.

^d^The Grasp Opportunity intervention removed all face-to-face components from CATCH-IT, including the motivational interview or brief advice component, and IPT modules were also excluded.

^e^OCD: obsessive compulsive disorder.

^f^COPE: Creating Opportunities for Personal Empowerment.

^g^Breathe: Being real, easing anxiety: tools helping electronically.

^h^Content covered in both interventions seems to be the same; however, Radomski et al 2020 [64] reported 6 modules and O’Connor et al 2020 [61] reported 8 modules.

^i^TFD: think, feel, do.

^j^Motivational interviewing was classified as a form of supported use.

### Methodological Quality

All 6 non-RCT studies met the methodological quality criteria for inclusion. Of the 22 RCTs included in the review, 9 (41%) were assessed as having a low ROB, 9 (41%) as having some concerns, and 4 (18%) as having a high ROB. All these studies were included in the analysis.

### Digital Self-administration Guidelines as Disclosed in Study Manuscripts

#### Overview

The aspects of digital self-administration extracted from the included studies are contained in Table 3 and Multimedia Appendix 2 [26,27,47-76]. Only 1 study (the study by O'Dea et al [62]) reported how instructions for appropriate use were relayed to young people. In this study, an animation was used to inform young people that they could use the intervention as they wished.

**Table 3:** Components of appropriate use described in studies or derived from study protocols or intervention features.

Study and year published	Components of appropriate use (part 1)					
	Recipients	Target condition or therapeutic goals	Amount of intervention completion to benefit	Duration and frequency of use	Sequence of content	Skill enactment
Berg et al [47], 2020	15-19 year olds with clinically significant anxiety, without or without comorbid depression (other comorbidities excluded)^a^	Anxiety and depression^a^	None provided	8-week access^a^, 1 module per week	Sequential via numbered modules^b^	None provided
Bevan Jones et al [48], 2020^c^	13-23 year olds with a history or risk of depression^a^	Depression	None provided	Access for a minimum of 2 months^a^. Instructed “they could use the program as they wished”	Nonsequential^d^	None provided
Calear et al [49,50], 2009 and 2013	12-17 year olds^a^	Prevent or decrease depression and anxiety	Completion of all 5 modules	5-week access with 1 module delivered each week^a^, each module 20-40 minutes	Sequential via controlled delivery of modules^a^	None provided
Clarke et al [51], 2009	18-24 year olds with a history of depression or risk of depression^a^	Depression^a^	None provided	32-week access^a^, unrestricted, self-guided use	None provided	Intervention guides user to create a personalized self-contract to increase the frequency of selected pleasant activities (eg, taking a relaxing bath, going to a restaurant all by yourself)^b^. Prompted to record activities every few days^b^
Fleming et al [52], 2012	13-16 year olds with probable depression; those with severe depression were excluded^a^	Depression^a^	None provided	5-week access^a^, 1-2 modules per week, each module approximately 30-minute duration	Sequential^b^	After each level, the digital guide reflected on how the learning could be applied in real life and set homework challenges^b^
Ip et al [53], 2016	13-17 year olds with mild or moderate depressive symptoms^a^	Reduce depressive symptoms (mild to moderate level) or prevent the onset of major depressive episodes.^a^ “Improve negative cognition, reduce negative behaviors, strengthen resiliency, and reinforce positive behaviors”	None provided	Access for the study period (12 months)^a^, use at anytime, anywhere	None provided	None provided
Jaycox et al [54], 2019	High school students with limited mental health resources^a^	PTSD^e^, anxiety, and depression^a^	None provided	1-2 chapters per week	Sequential^b^	Goal setting at the end of each module^b^
Kuosmanen et al [55], 2017	15-20 year olds^a^	Prevent depression; “aimed for young people who feel down, stressed or angry”	None provided	20-30–minute modules, completion of one module each week^a^	Sequential levels^b^	None provided
Lenhard et al [56], 2017	12-17 year olds with a primary OCD^f^ diagnosis^a^	OCD^a^	None provided	12 weeks access^a^	Sequential^b^	Young person and parent encouraged to complete ERP^g^ exercises together and report back to clinician
Lillevoll et al [57], 2014	Senior high school students^a^	Prevent and reduce depressive symptoms	None provided	45-60 minute modules, 6-7 week access^a^	Sequential via locked content^b^	None provided
Lucassen et al [58], 2020	12-19 year olds^a^	Prevent and treat depressive symptoms	None provided	Open access, modules take 30 minutes	Sequential levels^b^	Set challenges are provided to allow practice and facilitate skill generalization^b^
March et al [26], 2018	7-17 year olds with elevated anxiety^a^	Anxiety^a^	None provided	20-week access, 1 session each fortnight^a^	Sequential^b^	None provided
Melnyk et al [59], 2015	Freshman college students^a^	Depression and anxiety^a^	None provided	10-12–week access,^a^ 30-minute modules, 1 module per week	Sequential via locked content^b^	Weekly skill building homework assignments and goal setting logs
Merry et al [60], 2012	12-19 year olds with mild to moderate depressive symptoms^a^	Clinically significant depression^a^	None provided	4-7–week access^a^ 30-minute modules	Sequential^b^	“Virtual” guide,“ sets and monitors real-life challenges, equivalent to homework”^b^
O’Connor et al [61], 2020	13-17 year olds with a self-identified anxiety concern^a^	Anxiety^a^	None provided	8 weeks of website access^a^ instructed to use weekly	Sequential via numbered modules^b^	“Try Out feature, which outlined activities for the adolescent to choose to practice the module’s key concepts and skills”^b^
O’Dea et al [62], 2020^h^	12-16 year olds^a^	Depression and anxiety	Completion of all 4 character modules	4-week access^a^; self-paced	Nonsequential^b^	None provided
O'Kearney et al [63], 2009	High school girls aged 15-16 years^a^	Aims to reduce depression and vulnerability to depression	None provided	6-week access^a^; self-paced	Sequential^a^	None provided
Radomski et al [64], 2020	13-19 year olds with self-reported anxiety^a^	Aims to address mild to moderate anxiety symptoms	None provided	30 min each, complete one session per week	Numbered modules indicate sequential content^b^	Skill enactment prompted between modules^b^
Smith et al [65], 2015	Designed for adolescents with mild to moderate depression	Depression^a^	None provided	8-week access^a^, 30-40 min modules	None provided	“Designs own individualised homework based on specific technique”^b^
Spence et al [66], 2011	12-18 year olds meeting diagnostic criteria for social anxiety disorder, separation anxiety disorder, generalized anxiety disorder, or specific phobia^a^	Reduction in anxiety diagnostic status and severity^a^	Completion of all 10 modules	60 min modules, one module weekly	Sequential^b^	Responses to homework activities are accessed by therapist and feedback is provided via email^a^
Stallard et al [67], 2011	11-16 year olds with depression or anxiety assessed as suitable for CBT^a^	Depression and anxiety^a^	None provided	30-45 minute modules	Sequential via numbered modules^b^	“At the end of each session, participants are given a brief assignment to complete”^b^
Stasiak et al [68], 2012	13-18 year olds self-referred with probable or at risk of depression^a^	Depression^a^	Completion of all 7 modules	25-30 minute modules, complete between 4 and 10 weeks	Sequential via numbered modules^b^	Each module ends with a challenge (homework) for user to complete^b^
Stjerneklar et al [69], 2019	13-17 year olds meeting diagnostic criteria for an anxiety disorder^a^	“Reduce diagnostic severity and anxiety symptoms”	None provided	30-minute modules 14-week access^a^	Sequential via order^b^	“Each module contains homework practice tasks [users]... encouraged to complete”^b^
Tillfors et al [70], 2011	Adolescents (15-21 years) meeting diagnostic criteria for social anxiety disorder^a^	Social anxiety symptoms^a^	Completion of all 9 modules	9-week access,^a^ 1 module per week	None provided	None provided
Van Voorhees et al [71], 2009	Adolescents (14-21 years) at risk of depression (persistent subthreshold depression)^a^	“The intervention was intended to reduce multiple thoughts, behaviors, and interpersonal interactions thought to increase vulnerability for depressive disorders... And strengthen behaviors, thoughts and interpersonal relations thought to be protective against depressive disorders”	None provided	None provided	None provided	None provided
Van Voorhees et al [72], 2020; Gladstone et al [73,74], 2018 and 2020	13-18 year olds with elevated depression symptoms or a history of depression or dysthymia^a^, at clinically significant risk of depression but not with current depression	Preventing the onset of depressive episode^a^	None provided	15-20 min modules, 12-month access^a^	Sequential^a^	None provided
Whittaker et al [75,76], 2017 and 2012	Nondepressed years, 9-12 years; students (13-17 year old)^a^	“Prevention of the onset of depression”	A minimum of half the intervention completed	2 messages each day for 9 weeks^a^	Sequential^b^	None provided
Wuthrich et al 2012 [27]	14-17 year olds with diagnosed anxiety disorder^a^	Anxiety^a^	Not provided	30-minute modules; 12-week access^a^	Not provided	None provided

^a^Appropriate use derived from the trial protocol.

^b^Appropriate use derived from description of an intervention feature.

^c^All groups received weekly mail feedback via the study platform on exercises from the therapist. In addition, one group was invited to chat with the therapist in a 30-minute session.

^d^Appropriate use reported as a statement in the manuscript.

^e^PTSD: posttraumatic stress disorder.

^f^OCD: obsessive compulsive disorder.

^g^ERP: exposure and response prevention.

^h^All groups received standard digital CBT, designed to be neutral and straightforward. One group additionally received learning support that involved interactive features.

#### Intended Recipients and Target Condition

A total of 7% (2/28) of study manuscripts contained explicit statements of the intended intervention recipients: adolescents with mild to moderate depression [65] and adolescents with clinically significant risk of depression [72]. For all other manuscripts, intended recipients were derived from the participant inclusion and exclusion criteria. Of these, intended recipients were required to meet the diagnostic criteria for an anxiety disorder in 17% (5/28) of studies, were symptomatic (anxiety, depression, or both) in 32% (9/28) of studies, were at risk of developing depression in 17% (5/28) of studies (ie, symptomatic and/or history of depression), and were asymptomatic in 3% (1/28) of studies. Symptom level was not relevant to intended users in 21% (6/28) of studies (universal sample) and symptom details were not provided in 7% (2/28) of studies. A total of 39% (11/28) of study manuscripts described the intended target condition. For the remaining 43% (17/28) of studies, the intended target condition was derived from the study protocol (primary outcome measure). Depression was the target condition in 50% (14/28) of studies, anxiety in 32% (9/28) and both anxiety and depression in 17% (5/28). Finally, 7% (2/28) of studies provided statements on the intervention’s therapeutic goals; both of the goals were to reduce unhelpful cognitions and behaviors, consistent with a CBT approach.

#### Intended Degree of Completion, Duration and Frequency of Use, and Sequence of Use

A total of 17% (5/28) of studies stated that all intervention modules should be completed, and 3% (1/28) of studies stated that half the modules should be completed. The remaining 82% (23/28) of studies did not provide this information. Although not explicitly stated as intended use by the study authors, 10% (3/28) of studies examined *noncompleters* and *completers*. These concepts appeared to be derived post hoc for analytical purposes. Definitions of the latter included those who completed ≥4 modules out of 8 [64], ≥3 modules out of 5 [63], and more than one-third of all activities [50].

None of the studies explicitly stated the intended duration of intervention use. This information was derived from the duration of intervention access in 82% (23/28) of studies (range 4 weeks to 12 months); however, 17% (5/28) of studies did not provide any information on this (eg, duration between pre- and postintervention assessments). Intended frequency of use was stated in 60% (17/28) of studies and was derived from an intervention feature in 3% (1/28) of studies. Of these 18 studies, 9 (50%) recommended that users complete 1 module per week, 2 (11%) recommended up to 2 modules per week, 1 (5%) recommended 1 module per fortnight, and 1 (5%) recommended that users read 2 messages per day. A total of 21% (6/28) of studies stated that the frequency of intervention use was unrestricted. There were 36% (10/28) of studies that did not provide any information regarding the intended frequency of use. The intended sequence of content was derived from intervention features, including numbered modules, locked content, or module display, in 64% (18/28) of studies, from the study protocols of 11% (3/28) of studies (prescribed delivery of content), and was stated in the manuscript in 3% (1/28) of studies. Of these 22 studies, interventions were intended to be completed sequentially in 20 (90%) studies, with 2 (10%) allowing users unrestricted access to alternate between modules. A total of 27% (6/28) of studies provided no information on the intended sequence of the intervention content.

#### Intended Skill Enactment

A total of 50% (14/28) of studies described intervention features that prompted homework activities. The remaining 50% (14/28) provided no information regarding recommendations for skill enactment.

#### Adherence-Promoting Features

Regarding supported use, 17% (5/28) of interventions were autonomous, 42% (12/28) were supported, 21% (6/28) were both supported and intervention-led blended, and 10% (3/28) were intervention-led blended only. A total of 28% (8/28) of interventions used reminders (eg, email or SMS text message), 3% (1/28) implemented rewards (a snack upon completion of a module), and 21% (6/28) contained gamified concepts (eg, leveling up). Regarding interactive content, 82% (23/28) of studies described interventions that used interactive features, including 20 with interactive activities, 8 with quizzes, 7 with homework activities, and 18 with multimedia content. A total of 10% (3/28) of studies used tailoring, 10% (3/28) used personalized feedback, and 14% (4/28) used customization of visual features. None of the interventions were reported to contain peer-support features.

Only 10% (3/28) of studies have examined the influence of an adherence-promoting strategy on adherence to recommended use. Van Voorhees et al [71] examined supported use as an adherence promoter by combining the digital intervention with 3 in-person motivational interviewing sessions. These were found to significantly increase site visits, time in intervention, proportion of exercises completed, and number of characters typed into interactive exercises. Berg et al [47] evaluated 2 strategies: supported use via chat sessions with a therapist and learning support, which included short summaries, pedagogical pictures, videos, and quizzes. These strategies, alone or combined, did not lead to greater adherence when compared with the control condition. Finally, Lillevoll et al [57] evaluated the influence of personalized email reminders on adherence to MoodGYM compared with standard email reminders and no reminders. Neither form of reminder affected adherence to the intervention.

#### Core Therapeutic Components, Symptom Monitoring, and Accessing Crisis Support

A total of 14% (4/28) of studies reported core therapeutic components, including exposure therapy, exposure and response prevention therapy, behavioral therapy, and cognitive restructuring. The remaining 85% (24/28) of studies did not identify any. A total of 42% (12/28) of studies described intervention features for monitoring mood and assessing safety. Of these, 50% (6/12) of studies described symptom monitoring that occurred *every few days* or weekly. The remainder (6/12, 50%) did not provide any information regarding the frequency of mood monitoring. Furthermore, 28% (8/28) of study manuscripts described intervention features that assessed suicide risk and responded with helping-seeking information. The remaining 85% (24/28) of studies did not provide any information on when users were recommended to seek crisis support.

#### Reporting on Aspects of Intended Use

Of the 10 aspects of appropriate use identified to inform instructions for use (presented in Figure 1), no study manuscript reported on all 10 aspects. The average number of aspects reported was 6.29 (SD 1.33) with a range of 3 to 8. Descriptions of intervention features and study protocol details were relied upon heavily to infer aspects of appropriate use (Table 3 and Multimedia Appendix 2).

#### Measures of Adherence

The measures of adherence used in the included studies are presented in Table 4. To support a clearer interpretation of the results, adherence measures were coded as follows: module completion, intervention visits or log-ins, time spent in intervention, activity or homework completion and practice, and total content completion. Study attrition was not considered to be an appropriate measure of intervention adherence, as it was not possible to differentiate the noncompletion of study assessments from adherence to the intervention. Almost all the studies (24/28, 85%) measured module completion, which was reported as either mean (and SD) module completion or as percentage of the sample completing a defined number of modules (eg, 14/28, 50% of study manuscripts reported the percentage of the sample completing all modules). Moreover, 21% (6/28) of studies reported intervention visits or log-ins, either as the mean or as the percentage of the sample visiting on a defined number of occasions. Furthermore, 25% (7/28) of studies reported time spent in the intervention, either as the mean total intervention time or as the mean time spent per event (eg, visit or module). A total of 32% (9/28) of studies reported activity completion as either the mean, percentage of all activities completed, percentage of the sample that completed a set number of activities, or percentage who engaged in any of the activities. Just under half of all studies (13/28, 46%) reported >1 measure of adherence, indicating multidimensional operationalizations of the construct.

**Table 4:** Methods for measuring adherence to digital interventions and their association with depression and anxiety outcomes (n=28).

Study and year published	Measure of adherence						Association with depression outcome		Association with anxiety outcomes
	Module completion	Site or pp visits or log-ins	Time spent in intervention (min)	Activity, homework completion or practice	Total intervention completion (%)				
Berg et al [47], 2020	Mean 5.46/8 modules (SD 2.82); all modules=39.2%	NR^a^	NR	NR	39.20	NR		NR	
Bevan Jones et al [48], 2020^b^	NR	1-2 per week=21%, 1-2 per month=44%, 1-2 total=26%	Several hours=3% approximately 1 hour=29% approximately 30 minutes=54%, few minutes=20%, no visits=3%	NR	NR	NR		NR	
Calear et al [49,50], 2009 and 2013	Mean 3.16/5 modules >3 modules=62%; all modules=32.7%	NR	NR	15% of sample completed at least 20 of 29 exercises	32.70	0^c^		0^c^	
Clarke et al [51], 2009	NR	Mean web visits 8.5 (SD 14.2), median web visits 6, range web visits 0-111, page hits (mouse clicks)	Mean 115.1 (SD 176.1), median 52, range 0-1088	NR	NR	Minutes in intervention—Pos^d^, page hits—Pos, mean web visits 0		NR	
Fleming et al [52], 2012	>4 modules=81%; all modules=69%	NR	NR	NR	69	NR		NR	
Ip et al [53], 2016	Median 3 (IQR 5), all modules=10% (n=26/257)	NR	Median=39.3 (IQR=63.4)^e^	NR	10	Neg^f^		Neg	
Jaycox et al [54], 2019	Mean 6.37/7 modules	NR	NR	% video watched=63−89%	NR	NR		NR	
Kuosmanen et al [55], 2017	Mean 5/7 modules, >4 modules=87%, all modules=30%	NR	NR	55%-65% practiced (variety of skills, eg, thought monitoring, thought challenging, problem-solving)	30	NR		NR	
Lenhard et al [56], 2017	Mean 8.52/12 modules ^e^, 1-5 modules=97%, all modules=27%	NR	NR	NR	27	NR		0	
Lillevoll et al [57], 2014	1-2 modules=64%, >3modules=14%, all modules=3%	NR	NR	NR	3	NR		NR	
Lucassen et al [58], 2020	1 module=53.7%, 1-3 modules=44.9%, >4 modules=8.8%, 1-6 modules=50.01%, all 7 modules=3.7%	NR	<25 minutes per module	NR	3.70	NR		NR	
March et al [26], 2018	Mean 2.21/10 modules (SD 2.44); includes patient that did not start the intervention, no modules=21.65%, 1-2 modules=48.05%, >3 modules=30.31%	NR	NR	17.72/25	NR	NR		NR	
Melnyk et al [59], 2015	All modules=99%	NR	NR	NR	NR	NR		NR	
Merry et al [60], 2012	>4 modules=86%, all modules=60%	NR	NR	62% completed most or all homework challenges	60	NR		NR	
O’Connor et al [61], 2020	No modules=6%, all modules=36%	NR	NR	NR	36% completed all 8 modules (N=13/36)	NR		NR	
O’Dea et al [62], 2020	No modules=7.1%, all modules=60%	Mean 4.29, range 1-11	Per story: mean 5.55 min; overall: mean 19 min	NR	60	NR		NR	
O’Kearney et al [63], 2009	>3 modules=30% (n=20/67)	NR	NR	NR	NR	0^c^		NR	
Radomski et al [64], 2020	Mean 2.2/8 modules (SD 2.3), all modules=19.4%, >4 modules (adherers)=27.9%, <3 modules=72.1%, no modules=35.3%	NR	NR	NR	19.4	NR		0	
Smith et al [65], 2015	>4 modules=93%, all modules=86%	NR	NR	NR	86	NR		NR	
Spence et al [66], 2011	Mean 7.5/10 modules, all modules=39%	NR	NR	NR	39	NR		NR	
Stallard et al [67], 2011	All completed=85%	NR	NR	NR	85	NR		NR	
Stasiak et al [68], 2012	All completed=94%	NR	NR	NR	94	NR		NR	
Stjerneklar et al [69], 2019	Mean 5.4/8 modules, all modules=30%, >4 modules=69%	Mean 24.4, range 7-51	NR	Mean 74.4	30	NR		NR	
Tillfors et al [70], 2011	Mean 2.9/9 modules^e^, range 1-6	NR	NR	NR	NR	0		0	
Van Voorhees et al [71], 2009	NR	Percentage of sample visiting site at least once=84.1%^g^	Mean 121	Mean percentage of tasks completed=64%^g^, mean number of characters typed in tasks=2724	NR	NR		NR	
Van Voorhees et al [72], 2020; Gladstone et al [73,74], 2018 and 2020	Mean 3.4/14 modules (SD 4.7)	Days visited the site=3.7 (4.5)	Total time on website (min)=100.2 (143.1)	Characters typed=3071 (4572)	NR	0		NR	
Whittaker et al [75,76], 2017 and 2012	NR	NR	NR	Half of the messages viewed by 19% of sample^e,h^	NR	0		NR	
Wuthrich et al [27], 2012	All modules—98.4%	NR	NR	NR	98.4	NR		NR	

^a^NR: association was not reported.

^b^Time spent in intervention was average per visit [48].

^c^Association between adherence and outcomes determined by completer versus noncompleter analysis.

^d^Pos: positive association.

^e^Adherence measure used to determine the adherence-outcome association.

^f^Neg: negative association.

^g^Data were collated when more than one group received the intervention.

^h^Actual, rather than self-reported outcomes.

#### Association Between Adherence and Outcomes

The heterogeneity of adherence measures, the different statistical methods used to determine an association between adherence and outcomes, and incomplete reporting of results made it difficult to extract comparable data for a meta-analysis. Associations are, instead, reported descriptively. A total of 19 (67%) studies did not report on the association between adherence and symptom reduction. Of the remaining 8 studies, 6 (21%) examined the relationship between adherence to the intervention and treatment outcomes for depression. A total of 4 studies, 2 (7%) of which compared outcomes for completers and noncompleters (as defined by the authors), found no association between adherence and depression outcomes. Furthermore, 7% (2/28) of studies found an association between depression and adherence, but in opposing directions, such that depression decreased as time in intervention increased as per Ip et al [53], whereas Clarke et al [51] found that depression reduced when the total time spent in intervention decreased. Moreover, 14% (4/28) of studies examined the association between adherence and anxiety outcomes: 10% (3/28) found no association, and 3% (1/28) found a negative association between symptom reduction and total time spent in intervention, whereby more time in the intervention was associated with lower anxiety symptoms [53]. None of the studies that used multiple measures of adherence examined the independent associations of these measures with treatment outcomes.

## Discussion

### Principal Findings

This systematic review aimed to synthesize how appropriate use has been defined in digital CBT interventions for youth, how instructions for use are relayed to young people and reported in studies, and how adherence to appropriate use has been operationalized and measured. This review found that none of the included studies systematically described definitions of appropriate use, which were instead derived from the study protocol or intervention features. Despite all interventions being based on CBT, the derived definitions of appropriate use varied widely among studies, particularly in terms of intended recipients, frequency and duration of use, and use of adherence-promoting features. The only component of use with some consistency among studies was the sequence of use, with 71% (n=20) of the 28 studies using interventions intended to be used sequentially. Nearly all included studies neglected to disclose how the instructions or recommendations for appropriate intervention use were relayed to young people, with only 3% (1/28) of studies providing such information [62]. The results showed that most studies (24/28, 85%) operationalized adherence as the degree of intervention completion, with significant heterogeneity in how this was measured, regardless of how appropriate use was defined. The most consistently reported measure of adherence was the percentage of the sample that completed the entire intervention (14/28, 50%). There was little evidence of an association between degree of use and improved outcomes.

Appropriate self-administration of digital CBT is critical for achieving the therapeutic goals. Therefore, a lack of reporting on both definitions of appropriate use and how instructions for use are provided to users is somewhat surprising. Indeed, an understanding of how the intervention creators desired the intervention to be used by youth had to be derived from the study protocol and intervention features for almost all components of appropriate use, with the frequency of use the only component explicitly stated in studies. These findings suggest that inadequate consideration has been given to developing nuanced definitions of appropriate use. As early digital CBT interventions were direct translations of in-person CBT, this omission may stem from the assumption that engagement with CBT should be consistent regardless of the delivery mode (ie, full program completion). However, given the current innovative approaches to CBT delivery, particularly the movement away from sequential delivery and expectations of full program completion [38], it is imperative to investigate patterns of digital CBT use that produce the greatest improvements in defining appropriate use. There is also little evidence that young users of digital CBT for depression and anxiety are provided with adequate instructions for use. The impact of this on young people’s treatment uptake, engagement, response, and attitudes toward digital mental health care remains unknown. Poorly relayed instructions for appropriate use to young people, their guardians, and health professionals may contribute to poor intervention adherence in digital CBT. It is possible that more nuanced definitions of appropriate use and clearly delivered instructions on use could improve intervention adherence and ultimately have beneficial effects on the outcomes.

The resulting lack of guidance on how to operationalize and measure adherence is an additional consequence of poorly defined recommendations for appropriate use. Specifically, the degree to which actual use aligns with appropriate use can only be determined if the appropriate use is adequately defined. We found that adherence was most often operationalized as the degree of program completion, demonstrating a lack of correspondence among definitions of appropriate use, which varied widely, and operationalization of adherence. This indicates that definitions of appropriate use are either inadequate or not being used to operationalize adherence. This finding is also consistent with findings in the adult literature, where appropriate use is often undefined but nevertheless operationalized generically as *the more use, the better* [31], with significant heterogeneity in how the amount of use is measured [29,79,80]. Our conceptualization of the appropriate use of digital CBT is made up of 10 components (based on in-person CBT manuals) and is therefore multidimensional. This suggests that the measurement of adherence should be multidimensional and nuanced enough to capture adherence to each aspect of appropriate use. Taken together, adherence definitions and measures that provide a meaningful indication of the extent to which interventions are used appropriately are lacking, and require refinement in future studies.

We found little evidence to suggest an association between amount of use and symptom improvement. Most studies did not examine or report a dose-response effect for the intervention being evaluated. However, of those that did, most found no association between use and either depression or anxiety outcomes, regardless of the type of measure used. This finding demonstrates that the pervasive notion of *the more use, the better* is unsupported for digital CBT. A tenuous association between use and outcomes is consistent with findings reported in a recent meta-analysis of youth digital CBT interventions, which found no association between adherence (measured as the amount of use) and outcomes [25]. It is also somewhat consistent with a review of adult literature, which found that the association between use and depression outcomes depended on the usage measure used [29]. The lack of association between use and outcomes is, however, in contrast to in-person CBT interventions, where there are clear associations between intervention attendance and therapeutic benefits [81,82].

There are several possible explanations for the lack of association between adherence and outcomes. First, it is possible that measuring adherence as the degree of intervention completion or use does not capture the complexity to which a person may or may not adhere to various aspects of appropriate self-administration. Specifically, current one-dimensional measures of adherence are unlikely to quantify adherence to other components of appropriate use that may be more critical to intervention outcomes such as skill enactment or accessing crisis support. However, as digital CBT has been shown to be effective in well-controlled trials, outcomes are unlikely to be completely unrelated to intervention use [80,83]. Another explanation is that users simply stop using the intervention when sufficient benefits have been obtained. This point within the intervention would be expected to vary among individuals, so no association between the amount of intervention use and benefits would be evident. This notion is supported by a study that used graphical modeling to identify a subset of users who showed the greatest improvement in depressive and anxiety symptoms despite spending less time in the intervention than other groups of users [37]. Finally, some critical components of the intervention—the *active ingredients*— may drive the therapeutic response, and it is completion of these components, rather than completion of the whole intervention per se that is associated with outcomes. These explanations suggest that the current narrow definitions of appropriate use as the degree of intervention completion or use are too generic, and a more nuanced approach to the measurement of adherence is required.

**Figure 2:**
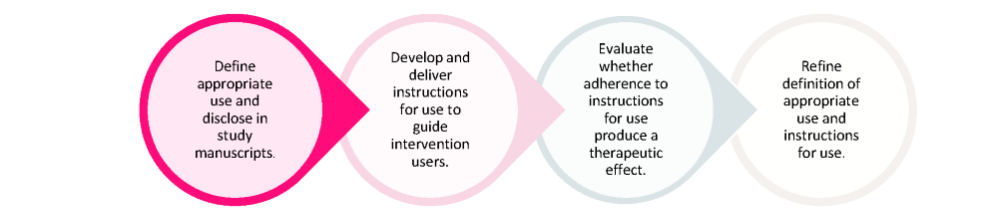
Process for defining, evaluating, and refining definitions of appropriate use and instructions for use.

Poorly defined recommendations for appropriate use may have several practical implications, including the reduced effectiveness of digital CBT because of inappropriate self-administration. Given the low uptake of evidence-based interventions [19,20], this presents a significant missed opportunity to improve young people’s mental health. It may also prevent the self-selection of interventions that are best suited to an individual’s circumstances and limit endorsement and dissemination by mental health professionals. Currently, there is little information in the scientific literature to guide the development of definitions of appropriate use or the disclosure of instructions for the use of digital CBT. In response to our findings and to rectify poorly defined appropriate use and generic operationalization of adherence, we developed a framework to support the development and disclosure of definitions of appropriate use and instructions for the self-administration of digital CBT. The results are presented in Table 5. It is based on the components of manualized CBT designed to guide clinicians in delivering in-person CBT and mapped onto definitions of intended use (the purpose of the product), indications for use (who will be using the product and why it will be used), and instructions for use (how to use the product) as defined in the medical arena [84]. The use of this framework to develop nuanced instructions for use could optimize the therapeutic benefits of interventions. Finally, we suggest an active evaluation of appropriate definitions and their refinement based on the outcomes of the evaluation. For example, a method to evaluate the extent to which the degree of completion, duration, and frequency of use incorporated in definitions of appropriate use produces therapeutic benefits is to conduct a survival analysis [85,86]. Comparing instructions for use related to degree of completion, duration, and frequency of use with those demonstrated to produce reliable and clinically significant reductions in symptoms, as determined by the survival analysis, will contribute to the refinement of the appropriate use definition. The proposed development, evaluation, and refinement of definitions of appropriate use and instructions for use is represented in Figure 2.

**Table 5:** Framework to develop definitions of appropriate use and operationalize adherence in digital CBT^a^.

Guideline			Description		Measure of adherence		Example—CBT intervention for specific phobia
**Indications for use**							
	Intended recipients	Who should use the intervention? Consider indications and contraindications for who should use the intervention.		Determine who is using the intervention		For 12-16 year olds with a specific phobia, without learning or developmental disorders	
	Intended target condition	What disorder has the intervention been designed to treat?		Determine symptomatic level of users		Specific phobia, for example, spider phobia	
**Intended use**							
	Therapeutic goals	Identify the specific outcomes the treatment aims to achieve.		N/A^b^		Reduce fear of spiders	
	Core therapeutic components (empirical or theoretical)	Identify intervention components that have been empirically or theoretically demonstrated to be associated with, or to mediate, improved outcomes.		Measure the extent to which core components are completed		Exposure hierarchy	
**Instructions for use**							
	Intended intervention completion	Define how much of the intervention should be completed.		What portion of users complete the required amount of the intervention		All modules of the intervention should be completed	
	Frequency and duration	What frequency and duration of use is required to produce therapeutic outcomes?		What portion of users engage at the required frequency and duration of use		Exposure to spiders for 3-6 months	
	Intended sequence	What pathway through the content will produce therapeutic outcomes?		Determine navigation through content and the portion of users engaging the required sequence (if relevant)		Content should be completed sequentially	
	Enactment of skills	What enactment or practice of skills is required beyond actual intervention use to achieve therapeutic outcomes?		Determine the extent to which users practice skills as required		Approach rather than avoid the next time when confronted with feared object or situation	
	Symptom monitoring	What level of symptom monitoring is required to produce therapeutic outcomes? What behaviors should outcomes of symptom monitoring produce?		Determine the extent to which users monitor symptoms as required (if relevant)		Anxiety symptoms are improving, move on to the next level of the exposure hierarchy (or cease treatment or trigger maintenance planning)	
	Access to crisis support	When should users be recommended to access crisis support and how will they access crisis support?		Determine the extent to which users access crisis support as suggested		N/A	
	Supported use	Consider whether self-guidance is unlikely to produce adequate adherence to appropriate self-administration.		Determine the extent to which support is accessed (if relevant)		Access to a clinician to construct exposure hierarchy	

^a^CBT: cognitive behavioral therapy.

^b^N/A: not applicable.

### Limitations

In this review, we limited our search to CBT for depression and anxiety, making it difficult to generalize our findings to other interventions or mental health conditions. Future studies could include other interventions and conditions to develop an understanding of how appropriate use and instructions for use have been defined and disclosed more broadly in digital health interventions. Moreover, our data failed to account for the possibility that appropriate use may have been defined or instructions for use provided to participants, but not documented in the study manuscripts or published protocols. However, consistently generic operationalization of adherence across studies suggests that this explanation is unlikely, and adequate and systematic documentation of these constructs is, nevertheless, required.

### Conclusions

This review has contributed to our understanding of how appropriate self-administration of digital CBT has been defined, relayed to users, measured, and associated with outcomes across various digital CBT interventions targeting depression and anxiety in young people. Overall, there is a lack of systematic reporting of nuanced definitions of appropriate use, and measures of adherence rarely provide adequate information on the degree of actual use corresponding to recommended appropriate use. There is little evidence that the degree of program completion or use is associated with intervention benefits. Our findings may, in part, explain low engagement in digital CBT; however, more work is required to better understand engagement with digital CBT and find ways to enhance it. A framework to assist in the development of guidelines for the self-administration of digital CBT has been provided.

## Appendix

Multimedia Appendix 1 Data extraction template.

Multimedia Appendix 2 Components of appropriate use.

## Abbreviations

CBT: cognitive behavioral therapy
PRISMA: Preferred Reporting Items for Systematic Reviews and Meta-Analyses
RCT: randomized controlled trial
ROB: Risk of Bias
SPARX: Smart, Positive, Active, Realistic, X-factor
