# Supplementary material for: Appropriate Use and Operationalization of Adherence to Digital Cognitive Behavioral Therapy for Depression and Anxiety in Youth: Systematic Review
Source: JMIR Ment Health. 2022 Aug 17;9(8):e37640. doi: 10.2196/37640 (PMC9434387; doi:10.2196/37640)
Supplement: Multimedia Appendix 2 [file mental_v9i8e37640_app2.docx]

| Study | Components of appropriate use | | | | | | | | | |  |  |  |  |
| --- | --- | --- | --- | --- | --- | --- | --- | --- | --- | --- | --- | --- | --- | --- |
|  | Recipients | | Target condition or therapeutic goals | Amount of intervention completion to benefit | Duration and frequency of use | Sequence of content | Skill enactment | Adherence-promoting features^a^ | Core therapeutic components | Symptom assessment and monitoring | Access crisis mental health support | Number of components reported |  |  |
|  | | | | | | | | | | | | | |  |
| Berg et al [47], 2020 | | 15-19 year olds with clinically significant anxiety, without or without comorbid depression (other comorbidities excluded)^a^ | Anxiety and depression^a^ | None provided | 8-week access,^a^ 1 module per week | Sequential via numbered modules^b^ | None provided | Support level, intervention-led blended^b^; interactive features^c^: activities, quizzes, audio or video files (multimedia), personalized feedback | None provided | None provided | None provided | 5 |  |  |
| Bevan Jones et al [48], 2020^c^ | 13-23 year olds with a history or risk of depression^a^ | | Depression | None provided | Access for a minimum of 2 months^a^. Instructed “they could use the program as they wished” | Nonsequential^d^ | None provided | Support level: autonomous tailoring; interactive features: activities, audio or video files (multimedia) | None provided | Mood monitor section in intervention^a^ | On the basis of mood monitoring responses, intervention will recommend user talks to someone or accesses “where to get help” section^a^ | 6 |  |  |
| Calear et al [49,50], 2009 and 2013 | 12-17 year olds^a^ | | Prevent or decrease depression and anxiety | Completion of all 5 modules | 5-week access with 1 module delivered each week^a^, each module 20-40 minutes | Sequential via controlled delivery of modules^a^ | None provided | Support level: supported; interactive features: activities, quizzes, homework, audio or video files (multimedia) | None provided | Premodule depression and anxiety quizzes track symptoms^a^ | None provided. | 7 |  |  |
| Clarke et al [51], 2009 | 18-24 year olds with a history of depression or risk of depression^a^ | | Depression^a^ | None provided | 32-week access,^a^ unrestricted, self-guided use | None provided | Intervention guides user to create a personalized self-contract to increase the frequency of selected pleasant activities (eg, taking a relaxing bath, going to a restaurant all by yourself)^b^. Prompted to record activities every few days^b^ | Support level: autonomous; interactive features: activities, personalized feedback; reminders | Cognitive restructuring and behavioral therapy “most curative element” | Mood and activity monitoring prompted every few days^a^ | None provided | 7 |  |  |
| Fleming et al [52], 2012 | 13-16 year olds with probable depression; those with severe depression were excluded^a^ | | Depression^a^ | None provided | 5-week access,^a^ 1-2 modules per week, each module approximately 30-minute duration | Sequential^b^ | After each level, the digital guide reflected on how the learning could be applied in real life and set homework challenges^b^ | Support level: supported; gamification; interactive features: activities, audio or video files (multimedia), customization | None provided | Premodule mood and safety check-ins by virtual guide^a^ | The guide “checks in” before each module and offers further help beyond the intervention^a^ | 8 |  |  |
| Ip et al [53], 2016 | 13-17 year olds with mild or moderate depressive symptoms^a^ | | Reduce depressive symptoms (mild to moderate level) or prevent the onset of major depressive episodes.^a^ “Improve negative cognition, reduce negative behaviors, strengthen resiliency, and reinforce positive behaviors” | None provided | Access for the study period (12 months)^a^, use at anytime, anywhere | None provided | None provided | Support level: supported; reminders | None provided | None provided | None provided | 4 |  |  |
| Jaycox et al [54], 2019 | High school students with limited mental health resources^a^ | | PTSD^f^, anxiety, and depression^a^ | None provided | 1-2 chapters per week | Sequential^b^ | Goal setting at the end of each module^b^ | Support level: supported; reminders; gamification; interactive features: activities, audio or video files (multimedia), tailoring | None provided | Selected chapters included assessment of anxiety, depression, and trauma symptoms^a^ | None provided | 7 |  |  |
| Kuosmanen et al [55], 2017 | 15-20 year olds^a^ | | Prevent depression; “aimed for young people who feel down, stressed or angry” | None provided | 20-30–minute modules, completion of one module each week^a^ | Sequential levels^b^ | None provided | Support level: supported; gamification; interactive features: activities, audio or video files (multimedia), customization | None provided | None provided | None provided | 5 |  |  |
| Lenhard et al [56], 2017 | 12-17 year olds with a primary OCD^f^ diagnosis^a^ | | OCD^a^ | None provided | 12 weeks access^a^ | Sequential^b^ | Young person and parent encouraged to complete ERP^g^ exercises together and report back to clinician | Support level: supported, intervention-led blended | Exposure and response prevention | None provided | None provided | 7 |  |  |
| Lillevoll et al [57], 2014 | Senior high school students^a^ | | Prevent and reduce depressive symptoms | None provided | 45-60 minute modules, 6-7 week access^a^ | Sequential via locked content^b^ | None provided | Support level: supported; interactive features: activities, quizzes, homework, audio or video files (multimedia), personalized feedback (for the tailored email condition only) | None provided | None provided | None provided | 5 |  |  |
| Lucassen et al [58], 2020 | 12-19 year olds^a^ | | Prevent and treat depressive symptoms | None provided | Open access, modules take 30 minutes | Sequential levels^b^ | Set challenges are provided to allow practice and facilitate skill generalization^b^ | Support level: autonomous; gamification; interactive features: activities, audio or video files (multimedia), customization | None provided | Premodule mood and safety check-ins by virtual guide^a^ | The guide “checks in” before each module and offers further help beyond the intervention^a^ | 8 |  |  |
| March et al [26], 2018 | 7-17 year olds with elevated anxiety^a^ | | Anxiety^a^ | None provided | 20-week access, 1 session each fortnight^a^ | Sequential^b^ | None provided | Support level: supported, intervention-led blended; reminders; interactive features: activities, quizzes | None provided | Anxiety scale integrated into the intervention at the beginning of the intervention and sessions 4, 7, and 10 | Automatic alert messages with help-seeking suggestions and resources were sent to participants if they reported clinically relevant anxiety scores^a^ | 7 |  |  |
| Melnyk et al [59], 2015 | Freshman college students^a^ | | Depression and anxiety^a^ | None provided | 10-12–week access,^a^ 30-minute modules, 1 module per week | Sequential via locked content^b^ | Weekly skill building homework assignments and goal setting logs | Support level: autonomous; interactive features: activities, homework, audio or video files (multimedia) | None provided | None provided | None provided | 6 |  |  |
| Merry et al [60], 2012 | 12-19 year olds with mild to moderate depressive symptoms^a^ | | Clinically significant depression^a^ | None provided | 4-7–week access^a^ 30-minute modules | Sequential^b^ | “Virtual” guide,“ sets and monitors real-life challenges, equivalent to homework”^b^ | Support level: supported; gamification; interactive features: activities, audio or video files (multimedia), customization | None provided | Premodule mood and safety check-ins by virtual guide^a^ | Participants who are not improving are prompted to seek help from their referring clinicians^a^ | 8 |  |  |
| O’Connor et al [61], 2020 | 13-17 year olds with a self-identified anxiety concern^a^ | | Anxiety^a^ | None provided | 8 weeks of website access^a^ instructed to use weekly | Sequential via numbered modules^b^ | “Try Out feature, which outlined activities for the adolescent to choose to practice the module’s key concepts and skills”^b^ | Support level: supported; reminders; tailoring; interactive features: activities, audio or video files (multimedia) | None provided | *“*Check-in feature which asked the youth to assess and rate their social-emotional functioning over the past week”^a^ | “Check-in/Check-out ratings that indicated thoughts of self-harm triggered a safety video and pop-up box, encouraging the adolescent to notify a parent or guardian of their thoughts and to seek immediate help”^a^ | 8 |  |  |
| O’Dea et al [62], 2020^h^ | 12-16 year olds^a^ | | Depression and anxiety | Completion of all 4 character modules | 4-week access^a^; self-paced | Nonsequential^b^ | None provided | Support level: autonomous reminders; interactive features: activities | None provided | None provided | “Participants who reported experiencing recent thoughts of death or of harming oneself were immediately displayed crisis contact details and encouraged to seek help from a trusted adult”^a^ | 7 |  |  |
| O'Kearney et al [63], 2009 | High school girls aged 15-16 years^a^ | | Aims to reduce depression and vulnerability to depression | None provided | 6-week access;^a^ self-paced | Sequential^a^ | None provided | Support level: supported; interactive features: activities, quizzes, homework, audio or video files (multimedia) | None provided | None provided | None provided | 5 |  |  |
| Radomski et al [64], 2020 | 13-19 year olds with self-reported anxiety^a^ | | Aims to address mild to moderate anxiety symptoms | None provided | 30 min each, complete one session per week | Numbered modules indicate sequential content^b^ | Skill enactment prompted between modules^b^ | Support level: supported; reminders; tailoring; interactive features: activities, audio or video files (multimedia) | None provided | Weekly check-in rating social-emotional functioning, including thoughts or harm or harm to others^a^ | None provided | 7 |  |  |
| Smith et al [65], 2015 | Designed for adolescents with mild to moderate depression | | Depression^a^ | None provided | 8-week access,^a^ 30-40 min modules | None provided | “Designs own individualised homework based on specific technique”^b^ | Support level: autonomous; interactive features: audio or video files (multimedia) | None provided | Reports on their homework and current mood^a^ | None provided | 6 |  |  |
| Spence et al [66], 2011 | 12-18 year olds meeting diagnostic criteria for social anxiety disorder, separation anxiety disorder, generalized anxiety disorder, or specific phobia^a^ | | Reduction in anxiety diagnostic status and severity^a^ | Completion of all 10 modules | 60 min modules, one module weekly | Sequential^b^ | Responses to homework activities are accessed by therapist and feedback is provided via email^a^ | Support level: supported, intervention-led blended; reminders; interactive features: activities, quizzes | None provided | None provided | None provided | 7 |  |  |
| Stallard et al [67], 2011 | 11-16 year olds with depression or anxiety assessed as suitable for CBT^a^ | | Depression and anxiety^a^ | None provided | 30-45 minute modules | Sequential via numbered modules^b^ | “At the end of each session, participants are given a brief assignment to complete”^b^ | Support level: intervention-led blended; interactive features: activities, quizzes, homework, audio or video files (multimedia) | None provided | None provided | None provided | 6 |  |  |
| Stasiak et al [68], 2012 | 13-18 year olds self-referred with probable or at risk of depression^a^ | | Depression^a^ | Completion of all 7 modules | 25-30 minute modules, complete between 4 and 10 weeks | Sequential via numbered modules^b^ | Each module ends with a challenge (homework) for user to complete^b^ | Support level: supported; reminders; interactive features: gamification, activities, audio or video files (multimedia) | None provided | None provided | “The program has build-in mood monitoring questions including risk of self-harm questions, which, if endorsed, resulted in a prompt on the computer suggesting that the young person see their counsellor for more help”^a^ | 8 |  |  |
| Stjerneklar et al [69], 2019 | 13-17 year olds meeting diagnostic criteria for an anxiety disorder^a^ | | “Reduce diagnostic severity and anxiety symptoms” | None provided | 30-minute modules 14-week access^a^ | Sequential via order^b^ | “Each module contains homework practice tasks [users]... encouraged to complete”^b^ | Support level: supported (parent and coach), intervention-led blended; interactive features: activities, homework, audio or video files (multimedia) | Psychoeducation, goal setting, cognitive restructuring, graded exposure | Weekly monitoring of symptom interference on functioning and mood^a^ | None provided | 8 |  |  |
| Tillfors et al [70], 2011 | Adolescents (15-21 years) meeting diagnostic criteria for social anxiety disorder^a^ | | Social anxiety symptoms^a^ | Completion of all 9 modules | 9-week access,^a^ 1 module per week | None provided | None provided | Support level: intervention-led blended; reminders; interactive features: quizzes | None provided | None provided | None provided | 5 |  |  |
| Van Voorhees et al [71], 2009 | Adolescents (14-21 years) at risk of depression (persistent subthreshold depression)^a^ | | “The intervention was intended to reduce multiple thoughts, behaviors, and interpersonal interactions thought to increase vulnerability for depressive disorders... And strengthen behaviors, thoughts and interpersonal relations thought to be protective against depressive disorders” | None provided | None provided | None provided | None provided | Support level: supported, intervention-led blended | None provided | None provided | None provided | 3 |  |  |
| Van Voorhees et al [72], 2020; Gladstone et al [73,74], 2018 and 2020 | 13-18 year olds with elevated depression symptoms or a history of depression or dysthymia^a^, at clinically significant risk of depression but not with current depression | | Preventing the onset of depressive episode^a^ | None provided | 15-20 min modules, 12-month access^a^ | Sequential^a^ | None provided | Support level: supported, intervention-led blended | None provided | None provided | None provided | 5 |  |  |
| Whittaker et al [75,76], 2017 and 2012 | Nondepressed years, 9-12 years; students (13-17 year old)^a^ | | “Prevention of the onset of depression” | A minimum of half the intervention completed | 2 messages each day for 9 weeks^a^ | Sequential^b^ | None provided | Support level: autonomous rewards; interactive features: audio or video files (multimedia) | None provided | None provided | None provided | 6 |  |  |
| Wuthrich et al 2012 [27] | 14-17 year olds with diagnosed anxiety disorder^a^ | | Anxiety^a^ | Not provided | 30-minute modules; 12-week access^a^ | Not provided | None provided | Support level: supported (parent), intervention-led blended; interactive features: activities, homework, audio or video files (multimedia) | “Strong focus on cognitive restructuring and graded exposure” | Not provided | Not provided | 5 |  |  |
